# Multiplex lateral flow immunochromatographic assay is an effective method to detect carbapenemases without risk of OXA-48-like cross reactivity

**DOI:** 10.1186/s12941-021-00469-0

**Published:** 2021-09-04

**Authors:** Hadas Kon, Shirin Abramov, Sammy Frenk, David Schwartz, Ohad Shalom, Amos Adler, Yehuda Carmeli, Jonathan Lellouche

**Affiliations:** 1 National Institute for Antibiotic Resistance and Infection Control, Ministry of Health, 6 Weizmann St., 6423906 Tel-Aviv, Israel; 2 Clinical Microbiology Laboratory, Tel-Aviv Sourasky Medical Center, Tel-Aviv, Israel; 3 Sackler Faculty of Medicine, Tel-Aviv University, Tel-Aviv, Israel; 4 The Miriam and Sheldon Adelson School of Medicine, Ariel University, Ariel, Israel

**Keywords:** NG-Test^®^ CARBA 5 assay, Cross reactivity, *Enterobacterales*, Carbapenemase, OXA-48-like, Carbapenem resistant

## Abstract

**Background:**

It is essential to detect carriers of carbapenemase-producing *Enterobacterales* in order to implement infection control measures. The objectives of this study was to evaluate the NG-Test^®^ CARBA 5 (CARBA 5) assay for detection of five carbapenemases and to assess the cross reactivity of other OXA-type carbapenemases with the OXA-48-like specific antibodies.

**Methods:**

A total of 197 *Enterobacterales* isolates were tested. To evaluate the cross reactivity, 73 carbapenem-resistant *A. baumannii*, harboring OXA-type variants, were tested. Polymerase chain reaction (PCR) served as gold standard for carbapenemase identification.

**Results:**

Excellent agreement was found between PCR and CARBA 5, for all but one isolate. The single false positive result (a *bla*_SME_ positive *S. marcescens* isolate) was incorrectly positive for *bla*_OXA-48_ by CARBA 5. No cross reactivity was observed. The sensitivity and specificity were 100.0% and 98.0%, respectively.

**Conclusions:**

The CARBA 5 assay is highly sensitive and specific and is recommended as a tool for the detection of the main carbapenemases of interest in clinical microbiology laboratories.

## Introduction

*Enterobacterales* are a common cause of both community-acquired and hospital-acquired infections [1, 2]. These bacteria can acquire genes encoding multiple antibiotic resistance mechanisms, including extended-spectrum β-lactamases (ESBLs), AmpCs, and carbapenemases [3]. The global rise of carbapenemase-producing *Enterobacterales* (CPE) presents an increasing threat to healthcare delivery and patient safety [4].

Rapid detection of patients colonized or infected by CPE is necessary in order to provide a fast and correct treatment protocol. The NG-Test^®^ CARBA 5 (CARBA 5) is a lateral flow immunoassay for carbapenemase detection, approved by the U.S Food and Drug Administration as of October 2019. CARBA 5 enables the detection of the five most prevalent carbapenemase enzymes (KPC, OXA-48-like, VIM, IMP, and NDM) through the use of specific antibodies [5].

Because lateral flow immunoassays are based on antigen–antibody interaction, there is a risk of cross reactivity [6]. In the CARBA 5 assay, cross reactivity may occur if an isolate harboring an OXA-type variant reacts incorrectly with the OXA-48-like target, leading to a false positive result. Several studies have evaluated the CARBA 5 assay and found high sensitivity and specificity rates [7–15], yet only two of these studies evaluated the cross reactivity of other OXA-type carbapenemases with the OXA-48-like specific antibodies [9, 14]. Here, we aimed to evaluate the CARBA 5 assay for detection of carbapenemases and to assess its specificity towards other OXA-type carbapenemases.

## Materials and methods

### Isolate selection

The isolates in our sample were selected in order to represent different years and hospitals from the collection of *Enterobacterales* isolates that were sent to the National Institute for Antibiotic Resistance & Infection Control in the years 2001–2017. An initial screening for carbapenemase identification was performed using polymerase chain reaction (PCR). We used a multiplex assay to detect *bla*_KPC_, *bla*_OXA-48-like_, *bla*_NDM_, *bla*_IMP_, *bla*_VIM_, and *bla*_IMI_ [16] and a simplex PCR to detect *bla*_SME_ [17]. The PCR results served as the gold standard to which the CARBA-5 assay was compared. Carbapenemase activity was assessed using the qualitative colorimetric β CARBA test (Bio-Rad, Marnes-la-Coquette, France), which is based on the change of color of an undisclosed chromogenic substrate in the presence of carbapenem-hydrolysing enzymes. The β CARBA test was conducted according to the manufacturer’s instructions.

The sample consisted of 197 unrelated, non-duplicate isolates from various sources (sputum n = 51, blood n = 74, urine n = 48, rectal n = 20, other n = 4). There were 19 (19/197, 9.6%) carbapenem-susceptible *Enterobacterales* isolates (CSE; meropenem MIC < 4 μg/ml) and 178 (178/197, 90.4%) carbapenem-resistant *Enterobacterales* (CRE; meropenem MIC ≥ 8 μg/ml). Of the 178 CRE isolates, 151 were identified as CPE: 47 harbored *bla*_KPC_, 40 *bla*_OXA-48-like_, 25 *bla*_NDM_, 19 *bla*_VIM_, 3 *bla*_IMI_, 1 *bla*_IMP,_ 1 *bla*_SME_ and 15 isolates harbored two or three carbapenemases. The other 27 CRE isolates (11 K*. pneumoniae*, 7 *E. coli*, 4 *Enterobacter* spp., 1 K*. oxytoca*, 1 *Proteus* spp., 1 *E. aerogenes* and 2 *Providencia* spp.) had meropenem MIC values of ≥ 8 μg/ml but no carbapenemase activity and none of the carbapenemases genes screened for in this study. They were classified as non-carbapenemase-producing CRE (Non-CP CRE). Meropenem MIC determination was performed twice using broth microdilution (BMD), according to Clinical and Laboratory Standards Institute (CLSI) guideline [18], at meropenem concentrations ranging from 0.5 to 64 mg/L. In case of discrepancy between duplicates, a third test was conducted. Susceptibility was determined using CLSI clinical breakpoint [19].

All isolates were stored at − 80 °C, sub-cultured aerobically at 35 ± 2 °C and transferred twice prior to testing.

### OXA-48-like target cross reactivity evaluation

To evaluate the cross reactivity with the OXA-48-like target, we tested 73 carbapenem-resistant *A. baumannii* (CRAB) isolates (meropenem MIC ≥ 8 μg/ml), all of them Ambler class D β-lactamases producers. *A. baumannii* isolates were identified by Vitek MS (BioMérieux, Marcy-l’Étoile, France) and identification was confirmed by PCR detection of *bla*_OXA-51-LIKE_ and *bla*_gyrB_ [20]. Isolates were tested by PCR for the *bla*_OXA-24_, *bla*_OXA-23_ and intrinsic *bla*_OXA-51-LIKE_ genes, as described previously [21]. PCR products were sequenced and OXA-type variants were determined using DNAMAN^®^ software version 7.0 (Lynnon Corporation, Pointe-Claire, Quebec) and the Basic Local Alignment Search Tool (BLAST, http://blast.ncbi.nlm.nih.gov). The CRAB isolates harbored *bla*_OXA-23_ (n = 6), *bla*_OXA-65_ (n = 6), *bla*_OXA-66_ (n = 11), *bla*_OXA-69_ (n = 2), *bla*_OXA-70_ (n = 4), *bla*_OXA-71_ (n = 11), *bla*_OXA-248_ (n = 8) and various OXA-type combinations (n = 25).

The CRAB isolates were stored at − 80 °C, sub-cultured aerobically at 35 ± 2 °C and transferred twice prior to testing.

### NG-Test CARBA 5 assay

The CARBA 5 assay (NG Biotech, Guipry, France) is based on the reaction of carbapenemases with labelled anti-carbapenemase monoclonal antibodies. The assay was performed using fresh colonies grown on CHROMagar™ mSuperCARBA™ (Hy Laboratories, Rehovot, Israel) or Mueller Hinton agar (Hy Laboratories), as recommended in the CARBA 5 instruction manual. One colony was suspending in five drops (150 μL) of extraction buffer. The bacterial suspension was homogenized by vortex and 100 μl was loaded into a nitrocellulose membrane. The suspension migrated through the membrane due to capillary force, and interacted with the corresponding anti-carbapenemase monoclonal antibodies immobilized on the membrane. Results were read by researchers blinded to the PCR results, to ensure unbiased interpretation, following 15 min of incubation time at room temperature. Results were considered positive if a red line appeared on the control region and on one or more of the test regions (KPC, OXA, VIM, IMP or NDM), indicating that the isolate carried one or more carbapenemases. Results were considered negative if a red line appeared only on the control line, indicating that the isolate did not carry any of the five carbapenemases.

Discrepancies between the CARBA 5 results and the PCR results were further investigated by genome sequencing**.**

### Genome sequencing

Isolates requiring investigation by genome sequencing were grown overnight on Brain Heart Infusion broth (Hy Laboratories) and DNA was extracted using the MagAttract HMW DNA Kit (Qiagen, Hilden, Germany). Purified DNA was sequenced using the Rapid Barcoding Sequencing (Ref. No. SQK-RBK004, Oxford Nanopore Technologies, Oxford, UK), on a MinION sequencing device (Oxford Nanopore Technologies). FastQ files were subjected to antibiotic resistance gene search using ResFinder K-mer alignment web interface [22]. Genome sequence was submitted to GenBank under BioProject No. SAMN17121013.

### Statistical analysis

Using PCR as the gold standard, we calculated the sensitivity and specificity of CARBA 5. We calculated 95% confidence intervals (CI) for each of these measures using VassarStats (http://vassarstats.net/prop1.html). If CARBA 5 detected the presence of a carbapenemase, but it differed from the carbapenemase detected by PCR, we considered the result a false positive (FP).

## Results

Confirmation of the initial screening for carbapenemase identification results was performed by PCR. Details of the *Enterobacterales* isolates are shown in Table 1. The β CARBA test confirmed the expression of carbapenemases for 148/151 CPE isolates; it did not detect carbapenemase activity in the three isolates harboring *bla*_IMI_, a gene for which some variants are not detectable by this test [23].

**Table 1:** Details of *Enterobacterales* isolates characterized by PCR

Organism tested	No. of isolates	KPC	NDM	OXA-48-like	VIM	IMP	IMI	SME	Combination	Non-CP CRE	CSE
	n (%)	n (%)	n (%)	n (%)	n (%)	n (%)	n (%)	n (%)	(n, %)	n (%)	
*K. pneumoniae*	77 (39.1)	32 (68.1)	6 (24.0)	16 (40.0)	8 (42.1)				OXA-48-like + NDM (2, 13.3)	11 (40.7)	
									KPC + VIM (1, 6.7)		
									VIM + NDM + OXA-48-like (1, 6.7)		
*E. coli*	52 (26.4)	5 (10.6)	7 (28.0)	21 (52.5)	2 (10.4)	1 (100.0)			KPC + NDM (7, 46.6)	7 (25.9)	
									OXA-48-like + NDM (2, 13.3)		
*E. cloacae*	13 (6.6)	2 (4.3)	3 (12.0)		6 (31.6)		1 (33.3)		KPC + NDM (1, 6.7)		
*Enterobacter* spp.	10 (5.1)	1 (2.1)		1 (2.5)	1 (5.3)		2 (66.7)		OXA-48-like + NDM (1, 6.7)	4 (14.9)	
*Citrobacter* spp.	6 (3.1)	3 (6.4)	3 (12.0)								
*K. oxytoca*	6 (3.1)	1 (2.1)	2 (8.0)	1 (2.5)	1 (5.3)					1 (3.7)	
*Proteus* spp.	4 (2.0)		2 (8.0)	1 (2.5)						1 (3.7)	
*E. aerogenes*	1 (0.5)									1 (3.7)	
*Providencia* spp.	3 (1.5)		1 (4.0)							2 (7.4)	
*S. marcescens*	3 (1.5)	2 (4.3)						1 (100.0)			
*K. ozonaea*	1 (0.5)				1 (5.3)						
*M. morganii*	1 (0.5)		1 (4.0)								
*R. planticola*	1 (0.5)	1 (2.1)									
CSE	19 (9.6)										19 (100.0)
Total	197 (100.0)	47 (23.9)	25 (12.7)	40 (20.3)	19 (9.6)	1 (0.5)	3 (1.5)	1 (0.5)	15 (7.6)	27 (13.8)	19 (9.6)

Non-CP CRE, non-carbapenemase-producing carbapenem-resistant *Enterobacterales*; CSE, carbapenem-susceptible *Enterobacterales*

The CARBA 5 assay accurately detected carbapenemase production in all 147 CPE isolates expressing a carbapenemase that the assay is designed to detect (KPC, OXA-48-like, VIM, IMP, and NDM); including isolates that were double or triple carbapenemase producers. Although results were read after 15 min, as per the manufacturer’s instructions, in most cases a positive result was obtained after 3–6 min. No false negative (FN) results were observed.

The CARBA 5 assay accurately identified as non-carbapenemase producers all 19 CSE and all 27 non-CP CRE. Additionally, the three CPE isolates carrying *bla*_IMI_ were identified as negative, as IMI is not targeted by CARBA 5. There was one discrepancy between PCR and CARBA 5 results: a *S. marcescens* isolate expressing a *bla*_SME_ gene according to PCR and *bla*_OXA-48_ according to CARBA 5. Genome sequencing confirmed the presence of *bla*_SME_ and the absence of a *bla*_OXA-48_ gene or one of its variants. We classified this error as a FP result. For this isolate, the red line on the CARBA 5 indicating a positive OXA-48 result, appeared only after 14 min and was faintly visible.

Initial *A. baumannii* isolates identification obtained by mass spectrometry was confirmed by OXA-51 and gyrB genes sequencing. Details of the 73 *A. baumannii* isolates harboring OXA-type variants are presented in Table 2. The isolates harbored one, two or three OXA-type variants simultaneously (OXA-23, -24/-40, -65, -66, -69, -70, -71, -82, -90, and -248). All 73 CRAB isolates harboring OXA-variants were correctly tested negative.

**Table 2:** Details of *A. baumannii* isolates harboring OXA-type variants

OXA-23	OXA-65	OXA-66	OXA-69	OXA-70	OXA-71	OXA-248	Combinations	Total
n (%)	n (%)	n (%)	n (%)	n (%)	n (%)	n (%)	(n, %)	n (%)
6 (8.2)	6 (8.2)	11 (15.1)	2 (2.7)	4 (5.4)	11 (15.1)	8 (11.0)	OXA-23 + OXA-66 (8, 11.0)	73 (100.0)
							OXA-71 + OXA-23 (11, 15.1)	
							OXA-23 + OXA-82 (2, 2.7)	
							OXA-65 + OXA-71 + OXA-23 (1, 1.4)	
							OXA-24 + OXA-71 + OXA-23 (2, 2.7)	
							OXA-24 + OXA-90 (1, 1.4)	

In our sample, CARBA 5 had 100.0% sensitivity (95% CI 97.4–100.0%) and 98.0% specificity (95% CI 89.5–99.6%).

## Discussion

Identification of carbapenemase genes can be done either by molecular tests, which detect the presence of a resistant carbapenemase gene, or by phenotypic tests, which detect the in vitro activity of carbapenemase enzymes [24, 25]. Molecular techniques, such as PCR, have several advantages, including accurate results and high sensitivity. However, molecular techniques cannot detect new or mutated genes and the implementation of these techniques requires skilled technicians [26]. Phenotypic tests, which are based on hydrolysis of a carbapenem (most often imipenem and meropenem), detect direct or indirect degradation products. Phenotypic tests include the Carba NP test [27], the modified carbapenem inactivation method (mCIM), the EDTA-modified carbapenem inactivation method (eCIM) and the rapid carbapenem inactivation method (rCIM) [28, 29]. Although the Carba NP test is recommended by the CLSI [19], a significant drawback of this test is the long preparation time required. Commercial derivatives of the Carba NP test are available and have simpler protocols, such as the Blue-Carba test [30]. The mCIM attain a high sensitivity and specificity, but the long turnaround time required (18–24 h) is a major limitation. Both the hydrolysis-based assays and the mCIM have limited ability to determine the exact carbapenemase enzyme, which might have important epidemiological and clinical implications.

In this study, we evaluated the performance of the CARBA 5 assay, a lateral flow immunoassay which is a simple, rapid and low-cost tool for carbapenemase detection. We found excellent agreement between the carbapenemase genes detected by PCR and the results of CARBA 5, for all but one isolate. The single FP result, obtained on a *bla*_SME_ positive *S. marcescens* isolate, was incorrectly positive for *bla*_OXA-48_ by the CARBA 5 assay. Bioinformatic analysis does not shed light on the reason causing this error.

Previous studies evaluating the CARBA 5 assay found a sensitivity ranging from 88 to 100% and a specificity varying between 95 and 100% [7–15, 31]. Three of these studies demonstrated 100% agreement [7, 12, 15]. Five evaluations reported FN errors obtained by isolates harboring IMP, NDM, VIM or OXA-48 [8–11, 13, 31]; whereas FP errors were reported in only three studies, attained from isolates carrying VIM or OXA-48-like genes [8, 10, 14]. In contrast to these evaluations, our study did not result in any FN errors, and obtained a single FP error.

The advantages of the CARBA 5 assay are numerous. CARBA 5 requires minimal preparation time and results are obtained within 15 min. The assay can be easily implemented in clinical microbiology laboratories and does not require external equipment or maintenance expenses. CARBA 5 enables detection of strains which express two or three carbapenemases simultaneously in a single test assay. It should be noted that the CARBA 5 assay cannot detect the IMI gene, therefore *E. cloacae* isolates with reduced susceptibility to meropenem need to be tested by other methods.

An alternative lateral flow immunoassay, RESIST-4 O.K.N.V. (Coris BioConcept, Gembloux, Belgium), is available for the detection of up to four carbapenemases. Several studies evaluating this test reported a specificity of 100% and a sensitivity ranging between 84 and 100%. Most errors were caused by isolates harboring *bla*_NDM_ (83–95% sensitivity for NDM) [11, 32–37], which may be due to subtypes of the target enzymes. The advantage of CARBA 5 is that it can detect an additional carbapenemase that RESIST-4 O.K.N.V. cannot: *bla*_IMP_.

Two prior studies evaluated the CARBA 5 for cross reactivity with the OXA-48-like target. Potron et al. evaluated cross reactivity on a small sample of 19 *Acinetobacter* spp., each harboring a single OXA-type variant (OXA-23, -24/-40, or -58) [9]. Boutal et al. tested cross reactivity using enterobacterial isolates that harbored OXA-1, -2, -9 or -10 [14]. Both of those studies, like our own, found no cross reactivity, demonstrating the low likelihood of an FP result generated by *Acinetobacter* in a heterogeneous culture.

Our study has some limitations. First, the *Enterobacterales* sample consisted of only one IMP positive strain; therefore, we cannot draw a clear conclusion for the CARBA 5 assay for the detection of IMP specifically. Second, the absence of carbapenemase activity for the non-CP CRE isolates was determined by PCR and the qualitative colorimetric β CARBA test; however, a definitive determination can only be complete by whole genome sequencing.

In conclusion, the CARBA 5 assay is highly sensitive and specific, rapid, and easy to implement in routine workflow. It is an accurate tool for the detection of the main carbapenemases of interest in clinical microbiology laboratories. This assay can serve as an alternative to PCR and is an effective tool for infection control and outbreak prevention.

## Data Availability

Not applicable.

## Abbreviations

CARBA 5: NG-Test^®^ CARBA 5
PCR: Polymerase chain reaction
ESBLs: Extended-spectrum β-lactamases
CPE: Carbapenemase-producing *Enterobacterales*
CSE: Carbapenem-susceptible *Enterobacterales* isolates
Non-CP CRE: Non-carbapenemase-producing CRE
CRE: Carbapenem-resistant *Enterobacterales*
CRAB: Carbapenem-resistant *A. baumannii*
CI: Confidence intervals
FP: False positive
FN: False negative
MCIM: Modified carbapenem inactivation method
ECIM: EDTA-modified carbapenem inactivation method
RCIM: Rapid carbapenem inactivation method
CLSI: Clinical and Laboratory Standards Institute
